# Transitionspsychiatrische Bedürfnisse und Identitätsentwicklung von Adoleszenten in Tirol

**DOI:** 10.1007/s40211-023-00477-w

**Published:** 2023-08-03

**Authors:** Kerstin Kunczicky, Ann-Christin Jahnke-Majorkovits, Kathrin Sevecke

**Affiliations:** 1 Medizinische Universität Innsbruck, Anichstr. 35, 6020 Innsbruck, Österreich; 2 Kinder- und Jugendpsychiatrie, Psychotherapie und Psychosomatik, A.ö. Landeskrankenhaus Hall, Milser Straße 10, Haus 6, 6060 Hall in Tirol, Österreich

**Keywords:** Transitionspsychiatrie, Transitionsbereitschaft, Transitionsbedürfnisse, Identitätsentwicklung, Transitional Psychiatry, Readiness for transition, Transition needs, Identity development

## Abstract

**Grundlagen:**

Durch die strukturelle Trennung der kinder- und jugendpsychiatrischen und der erwachsenenpsychiatrischen Behandlung ist ein Wechsel zwischen diesen beiden System mit Erreichen des 18. Lebensjahres vorgesehen. Vor dem Hintergrund der Bedeutung eines organisierten Übergangs (Transition) wurden Patient:innen im Transitionsalter ab 17 Jahren zu ihren Transitionsbedürfnissen und Aspekten ihrer Identitätsentwicklung befragt. Dadurch sollen zukünftige Verbesserungen der Versorgungsstrukturen in dieser sensiblen Phase abgeleitet werden.

**Methodik:**

Mit Hilfe des Transition Readiness and Appropriateness Measure (TRAM) wurden die transitionsspezifischen Bedürfnisse der 17- bis 24-jährigen Patient:innen (*N* = 39) der Abteilung für Kinder- und Jugendpsychiatrie, Psychosomatik und Psychotherapie in Hall in Tirol befragt. Außerdem wurde anhand des *Assessment of Identity Development in Adolescence *(AIDA) der Zusammenhang der Identitätsentwicklung und der Transition untersucht.

**Ergebnisse:**

Knapp 80 % der befragten Patient:innen gaben einen weiteren Versorgungsbedarf an. Als Barrieren bezüglich der Transitionsbereitschaft wurden „*patientenbezogene Faktoren*“ als auch die „*Unterstützung durch die Familie*“ beschrieben. Zwischen der Identitätsentwicklung und der Transitionsbereitschaft bzw. dem -bedarf wurde ein signifikanter Zusammenhang gefunden (r = 0,431, *p* < 0,01), wobei dieser auf den Transitionsbedarf bezogen, stärker ausgeprägt war (r = 0,821 *p* < 0,01). Außerdem gaben die Betroffenen an, häufig und stark durch Stress belastet zu sein, 45 % berichteten von selbstverletzendem Verhalten, 48 % von Suizidgedanken bzw. -verhalten in den letzten sechs Monaten. Hinsichtlich der Psychopathologie zählten Ängste und Depressionen zu den häufigsten Symptomen. In der Beeinträchtigung des Funktionsniveaus war vor allem der Bereich „Beziehungen“ am stärksten betroffen.

**Schlussfolgerungen:**

Die Untersuchung liefert erste Erkenntnisse zu den transitionsspezifischen Merkmalen und Bedürfnissen von Patient:innen im Transitionsalter. Eine Integration von standardisierten Messinstrumenten in institutionelle Versorgungssysteme, die die transitionsbezogenen Bedürfnisse, die Transitionsbereitschaft und den -bedarf von jungen Menschen im Transitionsalter individuell erfassen, kann eine zielgerichtete und bedürfnissgerechte Behandlung bzw. Transition erleichtern. Auch eine interdisziplinäre Zusammenarbeit der Kinder- und Jugendpsychiatrie und der Erwachsenenpsychiatrie sowie eine kontinuierliche Überführung der therapeutischen Beziehungen im Transitionsprozess sollten standardisiert gewährleistet werden.

## Abstract (en)

**Background:**

Due to the structural separation of child and adolescent psychiatric treatment and adult psychiatric treatment, a switch between these two systems upon the age of 18 is necessary. Considering the importance of an organized transition, patients from an age of 17 were asked about their transition needs and aspects of their identity development. Thus future improvements in the treatment structures in this sensitive phase should derive.

**Methods:**

With the help of the Transition Readiness and Appropriateness Measure (TRAM), the transition-specific needs of 17 to 24-year-old patients (*N* = 42) at the Clinic for Child and Adolescent Psychiatry, Psychosomatics and Psychotherapy in Hall in Tirol were surveyed. In addition, the connection between identity development and transition was examined using the Assessment of Identity Development in Adolescence (AIDA).

**Results:**

Almost 80% of the patients stated that they needed further care. ‚Patient-related factors‘ and ‚family support‘ were described as barriers to the willingness to transition. A significant connection was found between identity development and the willingness or need for transition (r = 0.431, *p* < 0.01), although this was more pronounced in relation to the need for transition (r = 0.821 *p* < 0.01). In addition, those affected stated that they were frequently and severely burdened by stress, 45% reported self-injurious behavior, 48% suicidal thoughts or behavior in the last six months. In terms of psychopathology, anxiety and depression were among the most common symptoms. In terms of functional impairment, the ‚relationships‘ area was the most affected.

**Conclusions:**

The study provides initial insights into the transition-specific characteristics and needs of patients in transition age. The integration of standardized measuring instruments in institutional care systems, which individually record the transition-related needs, the willingness and need of young people in transition age, can facilitate targeted and needs-based treatment or transition. Interdisciplinary cooperation between child and adolescent psychiatry and adult psychiatry as well as a continuous transfer of the therapeutic relationships in the transition process should also be guaranteed.

## Hintergrund

Der Begriff der Transition umfasst einen multidimensionalen Prozess mit lebensverändernden Ereignissen. Damit ist einerseits die sensible Phase des Übergangs vom Jugend- ins Erwachsenenalter gemeint, andererseits bedient sich die Medizin der Begrifflichkeit im Sinne eines Transfers von einer jugendpsychiatrischen hin zur erwachsenenpsychiatrischen Versorgung [1]. Diese Lebensphase im Alter zwischen 18 und 30 Jahren, die auch als *Emerging Adulthood* definiert wird, ist durch ein hohes Maß an Instabilität und demografischen Wandel und ein damit zusammenhängendes Auftreten von psychischen Störungen gekennzeichnet [2, 3]. Empirischen Studien zufolge sind 50 % aller psychischen Erkrankungen im Alter von 14 Jahren und 75 % im Alter von 24 Jahren manifest [4]. Etwa 23,9 % der österreichischen Kinder und Jugendlichen im Alter von 10 und 18 Jahren leiden an einer psychischen Erkrankung [5]. Darüber hinaus müssen Jugendliche in dieser Phase die von Erikson (1968) [6] in seinem Stufenmodell benannte Krise der Ich-Identität vs. Ich-Identitätsdiffusion lösen, die unter Umständen zu einer Identitätskrise aufgrund der zunehmenden Diskrepanz der Selbst- und Fremdwahrnehmung führen kann [7]. Für Patient:innen in kinder- & jugendpsychiatrischen Einrichtungen, die sich mit anhaltenden und schweren psychischen Störungen präsentieren, bedeutet das Erreichen ihrer Volljährigkeit zugleich ein Wechsel in ein Gesundheitssystem für Erwachsene. Dieser Behandlungsübergang findet in den meisten EU-Ländern, so auch in Österreich, häufig unstrukturiert und mit Unterbrechungen in der Versorgung statt [8, 9]. Negative Auswirkungen auf Krankheitsverläufe und Prognosen der Betroffenen können die Folge sein [4]. Laut der britischen TRACK-Studie (*Transition from CAMHS to Adult Mental Health Services*) erfahren nur ca. 5 % der betroffenen Jugendlichen eine optimale Transition [10]. Eine fehlende Kommunikation zwischen den Behandler:innen sowie eine Überforderung der Jugendlichen durch die zunehmende Selbstverantwortung verstärkt den ohnehin schwierigen Übergang und mündet in häufige Behandlungsabbrüche [11].

Da die Verbesserung der Transitionspsychiatrie in den EU-Ländern derzeit einen hohen Stellenwert einnimmt, wurde von der Europäischen Union ein länderübergreifendes Forschungsprojekt zu diesem Thema realisiert, die sogenannte Milestone Study [12]. Das Ziel der Studie bestand darin, eine organisierte Transition durch die Entwicklung spezifischer Messinstrumente (TRAM: Transition Readiness and Appropriatness Measure und TROM: Transition Related Outcome Measure) und Trainingsmöglichkeiten für Mitarbeiter:innen sowie die Langzeitverfolgung von ehemaligen kinder- und jugendpsychiatrischen Patient:innen zu fördern. Zu diesem Zweck wurde in einem Zeitraum von 2014 bis 2019 das in der Milestone-Studie entstandene Modell einer organisierten Transition in einer prospektiven Kohortenstudie an über 1000 Jugendlichen sowie 900 Eltern und Mitarbeitern überprüft [12]. Dabei konnte gezeigt werden, dass sich die Symptome der Jugendlichen, bei denen eine organisierte Transition durch Rückmeldung der Transitionsbereitschaft durchgeführt wurde, schneller besserten als die von den Jugendlichen aus der Kontrollstichprobe mit dem üblichen Vorgehen [13].

Die Umfrage von Pollak, Kapusta, Diehm, Plener und Skala [14], konnte zeigen, dass eine unorganisierte Transition auch in Österreich von 98,8 % der befragten Kliniker:innen als ungünstig bewertet wird, 83,7 % würden sich eine Verbesserung der Kommunikation und Zusammenarbeit an den Schnittstellen wünschen und 65,1 % würden eine Erweiterung der kinder- und jugendpsychiatrischen Behandlung bis zum 24. Lebensjahr befürworten.

Vor diesem Hintergrund beschäftigt sich die vorliegende Studie nun mit der Frage, welche transitionspsychiatrischen Merkmale und Bedürfnisse betroffene Adoleszenten in Österreich aufweisen und welche zukünftigen Behandlungsstrategien im Bereich der Transitionspsychiatrie daraus abgeleitet werden können. Da der Aufbau einer stabilen Identität zu den zentralen Entwicklungsaufgaben von Adoleszenten zählt [1–3, 6, 15], wurde darüber hinaus erstmalig untersucht, inwiefern es Zusammenhänge zwischen der Identitätsentwicklung und der Transitionsbereitschaft bzw. des -bedarfs von Adoleszenten in der Transitionsphase gibt. Die Hypothesen dazu lauteten wie folgt: 1. Eine geringe Transitionsbereitschaft von Patient:innen im Alter von 17 bis 24 Jahren steht im Zusammenhang mit einer Identitätsdiffusion. 2. Ein hoher Transitionsbedarf von Patient:innen im Alter von 17 bis 24 Jahren steht in Zusammenhang mit einer diffusen Identitätsentwicklung.

## Methoden

### Vorgehen

**Abb. 1:**
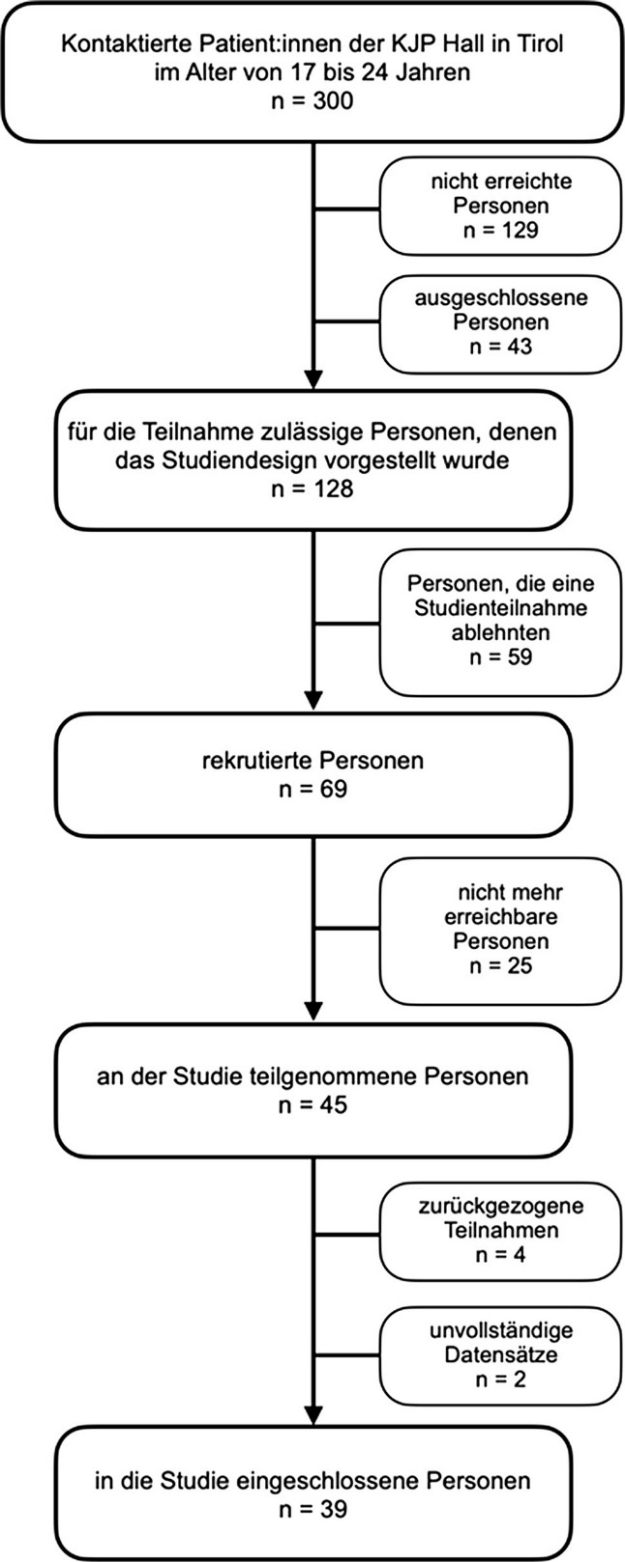
Flowdiagramm der Teilnehmer:innenrekrutierung

In einem Telefongespräch wurden von Januar 2021 bis einschließlich Oktober 2022 300 Patient:innen aus dem Patient:innenverwaltungsprogramm KIS anhand der Einschlusskriterien (kinder- und jugendpsychiatrische Behandlung an der Abteilung für Kinder- und Jugendpsychiatrie, Psychotherapie und Psychosomatik Hall in Tirol, Alter 17 bis 24 Jahre, ausreichende Deutschkenntnisse und ausreichende kognitive Fähigkeiten, um die Fragebögen zu beantworten) sowie der Ausschlusskriterien (keine kinder- und jugendpsychiatrische Behandlung, erwachsenenpsychiatrische Behandlung, fehlendes Einverständnis der Adoleszenten oder Erziehungsberechtigten und fehlende Fähigkeiten die Fragebögen zu bearbeiten z. B. starke Intelligenzminderung, unzureichende Kenntnisse der deutschen Sprache) rekrutiert. Von den 300 kontaktierten Personen waren 129 nicht erreichbar, 43 Personen erfüllten nicht die Einschlusskriterien. Dabei musste eine Person aufgrund von mangelnden Deutschkenntnisse ausgeschlossen werden. Von den restlichen 128 Personen gaben 59 kein Einverständnis gegenüber der Studienteilnahme und 25 erklärten sich zwar zur Teilnahme bereit, waren aber zum Zeitpunkt der Befragung telefonisch nicht erreichbar. Schlussendlich durchliefen 45 Personen die Fragebogenbatterie. Der Stichprobenumfang wurde aufgrund unvollständiger Datensätze (N = 2) und zurückgezogener Teilnahmen (N = 4) auf N = 39 Teilnehmer:innen reduziert (siehe Abb. 1). Die Befragung wurde über ein Online-Survey unter telefonisch gestützter Anleitung durchgeführt. Alle Patient:innen und bei minderjährigen zusätzlich deren Obsorgeberechtigten unterzeichneten vor der Teilnahme an der Untersuchung eine Einwilligungserklärung nach einer ausführlichen Patienteninformation. Zur Untersuchung der Stichprobe wurden die soziodemografischen Merkmale sowie die aktuelle bzw. frühere Behandlungssituation, die standardisierten Fragebögen TRAM (aus der Milestone-Studie) und AIDA (Assessment of Identity Development in Adolescence) verwendet. Die Ethikkommission der Medizinischen Universität Innsbruck erteilte die Bewilligung für die Durchführung und Publikation der vorliegenden Studie (EK Nr.: 1444/2020 vom 17.12.2020).

### Beschreibung der Stichprobe

**Abb. 2:**
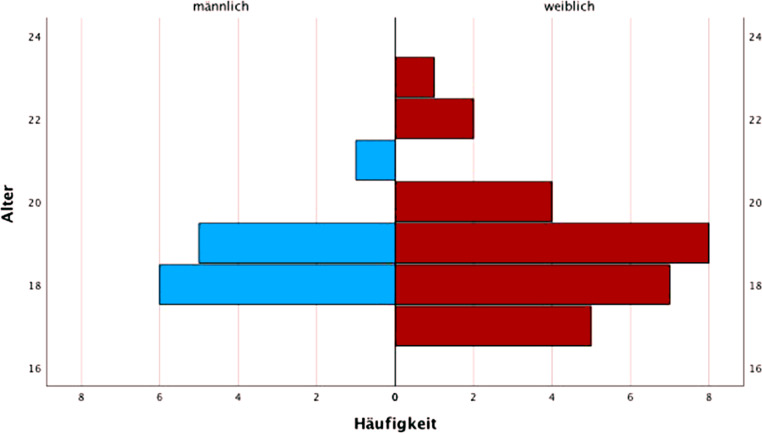
Häufigkeit des Alters in der Geschlechterverteilung

Von den insgesamt 39 Teilnehmer:innen waren 27 Personen (69,2 %) weiblich und 12 (30,8 %) männlich mit einem Alter zwischen 17 und 23 Jahren. Das Durchschnittsalter zum Zeitpunkt der Erhebung betrug 18,8 Jahre (M = 18,82; SD = 1,39). Aus der Abb. 2 kann die Häufigkeit des Alters der männlichen und weiblichen Teilnehmenden entnommen werden.

Aus der Tab. 1 gehen die soziodemografischen Merkmale der Teilnehmer:innen hervor. Die überwiegende Mehrheit hatte einen Hauptschulabschluss (N = 21; 53,8 %) erworben, war Schüler:in (N = 14; 35,9 %) oder berufstätig (N = 13; 33,3 %). Außerdem lebte der Großteil der Studienpopulation (N = 23; 59,0 %) bei den Eltern und in einem Dorf bzw. einer ländlichen Region (N = 21; 53,8 %). Fast 60 % (N = 18; 59,0 %) berichteten Single zu sein und in der Mehrheit der Fälle (N = 18; 46,2 %) lebten die Teilnehmer:innen mit ihren Eltern in einem Haushalt.

**Tab. 1:** Überblick der soziodemografischen Merkmale

	N	%
*Höchst abgeschlossener Bildungsstand*		
Matura	11	28,2
Fachschulreife	6	15,4
Hauptschulabschluss	21	53,8
Keinen	1	2,6
*Berufliche Tätigkeit*		
Schüler:in	14	35,9
Student:in	6	15,4
Auszubildene:r	6	15,4
Berufstätigkeit	13	33,3
*Wohnsituation*		
Allein	8	20,5
Bei den Eltern	23	59,0
In der Wohngemeinschaft	7	17,9
In einer Jugendhilfe-Einrichtung	1	2,6
*Wohnort*		
Stadt (über 100.000 Einwohner)	12	30,8
Kleinstadt (10.000–20.000)	6	15,4
Dorf/ländliche Region	21	53,8
*Aktueller Beziehungsstatus*		
Ledig	23	59,0
In einer Partnerschaft	15	38,5
Verheiratet	1	2,6
Getrennt/geschieden	0	0,0
*Aktueller Beziehungsstatus der Eltern*		
Leben zusammen	18	46,2
Getrennt/geschieden	16	41,0
Nie zusammen gelebt	3	7,7
Durch den Tod getrennt	2	5,1

**Abb. 3:**
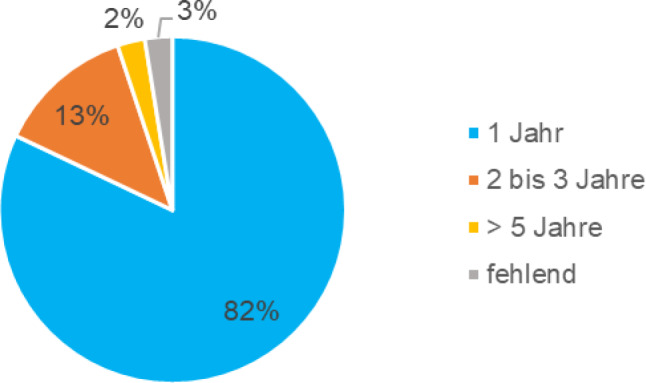
Behandlungsdauer in der psychiatrischen Versorgung

Zum Erhebungszeitpunkt erhielten 72 % (N = 28; 71,8 %) keine psychiatrische Behandlung. Die restlichen Teilnehmer:innen waren aktuell in ambulanter (N = 6; 15,4 %), stationärer (N = 1; 2,6 %) und/oder tagesklinischer (N = 1; 2,6 %) kinder- und jugendpsychiatrischer Versorgung. Mehr als 97 % hatten vorher eine kinder- und jugendpsychiatrische Behandlung. Dabei war die Versorgung bei 46,2 % (N = 18) ambulant, bei 51,3 % (N = 20) stationär und/oder bei 12,8 % (N = 5) tagesklinisch. Die durchschnittliche Behandlungsdauer war 206,0 Tage (M = 206,03; SD = 378,09). Die Dauer der Behandlung kann aus dem Kreisdiagramm in der Abb. 3 abgelesen werden.

### Erhebungsinstrumente

Die Transition Readiness and Appropriateness Measure (TRAM, [16]) besteht aus 58 Items und 8 verschiedene Sub-Skalen: (A) Symptome, in ihrer Häufigkeit und Schwere, (B) allgemeiner Krankheitsstatus, (C) allgemeine Beeinträchtigung des Funktionsniveaus, (D) Risikofaktoren, in ihrer Häufigkeit und Schwere, (E) Faktoren, welche die Symptomatik beeinflussen, (F) Faktoren des Gesundheitssystems, (G) Barrieren, die ein Funktionieren der Transition behindern und (H) andere Lebensveränderungen. Dieser wurde in der Untersuchung von Santosh et al. [16] auf seine Validität und Reliabilität geprüft und ergab in Cronbachs α ≤ (umgekehrt) 0,70 eine annehmbare Reliabilität und gute bis moderate psychometrische Eigenschaften (für die Domänen A, C, D, E und G: α = 0,804; α = 0,869; α = 0,735; α = 0,554 und α = 0,616) [16].

Das *Assessment of Identity Development in Adolescence* (AIDA, AIDA19+, [17]) dient bei 12- bis 18-Jährigen zur Unterscheidung einer gesunden Identitätsintegration, Identitätskrise und einer klinisch auffälligen Identitätsdiffusion und enthält 58 Items mit einer 5‑stufigen Likert-Skala von 0 = nein, 1 = eher nein, 2 = teils/teils, 3 = eher ja, 4 = ja. Zu den Hauptskalen zählen Kontinuität bzw. Diskontinuität und Kohärenz bzw. Inkohärenz. Jede Hauptskala besteht aus drei Subskalen: selbstbezogen (Stabilität in den Zielen bzw. konsistentes Selbstbild), sozialbezogen (Stabilität in den Rollen bzw. Autonomie) und reflexionsbezogen (emotionales Selbsterleben bzw. kognitives Selbsterleben) [18]. Der AIDA wurde als ein zuverlässiges Frageninventar in mehreren Validierungsstudien anerkannt [18–20]. Der AIDA +19 wurde bei den Teilnehmer:innen ab dem 19. Lebensjahr angewandt.

### Statistische Analysen

Sämtliche Daten wurden mittels Statistikprogramm IBM SPSS Statistics 28 (IBM Corporation, Armonk, NY, USA) ausgewertet. Dabei kamen eine deskriptive Statistik, Häufigkeiten und Mittelwerte mit Standardabweichungen als auch der Korrelationskoeffizient nach Pearson zur Anwendung. Die Normalverteilung einer Stichprobe wurde bei einer Gruppengröße von N > 30 nach dem zentralen Grenzwertesatz angenommen [21]. Wenn die Beantwortung von ganzen Skalenbereichen (z. B. für den TRAM die Sub-Skala A) ausblieb, musste der Stichprobenumfang entsprechend minimiert werden. Gleiches passierte bei > 8 fehlenden Angaben im AIDA.

## Ergebnisse

### Transition Readiness and Appropriateness Measure (TRAM)

Die Tab. 2 dient zur Veranschaulichung der errechneten Punkte, welche die Teilnehmenden im Durchschnitt in den einzelnen Sub-Skalen und den dazugehörigen Teilbereichen im TRAM erzielten. Zusätzlich wird die maximal erreichbare Punktezahl dargestellt.

**Tab. 2:** Ergebnisse des TRAM

	M	SD	Max. Punktezahl
*Sub-Skala A – Symptomatik*	38,18	20,25	110
Internalisierende Symptomatik	26,97	14,50	70
Weiblich	27,93	14,29	70
Männlich	24,83	15,37	70
Externalisierende Symptomatik	11,21	8,42	40
Weiblich	11,21	8,42	40
Männlich	13,33	7,78	40
*Sub-Skala C – Funktionsniveau*	11,46	7,82	40
Aktivitäten des täglichen Lebens	3,28	3,12	16
Beziehungen	8,18	5,31	24
*Sub-Skala D – Risikofaktoren*	14,54	10,29	60
Internale Risikofaktoren	11,03	7,01	30
Externale Risikofaktoren	3,54	4,72	30
*Sub-Skala E – Symptomatik beeinflussenden Faktoren*	2,51	1,27	7
Wiederauftreten der Erkrankung	2,44	1,29	5
Somatische Krankheitsfaktoren	0,08	0,27	2
*Sub-Skala G – Transitionsbarrieren*	8,00	3,83	27
Patientenbezogene Faktoren	3,92	2,43	15
Unterstützung durch die Familie	2,72	1,52	6
Behandlung	1,36	1,48	6
*Appropriateness of Transition* (Sub-Skala A, B, C, D + E)	68,72	36,36	275

*M* Mittelwert, *SD* Standardabweichung;

#### Transitionsbarrieren

**Abb. 4:**
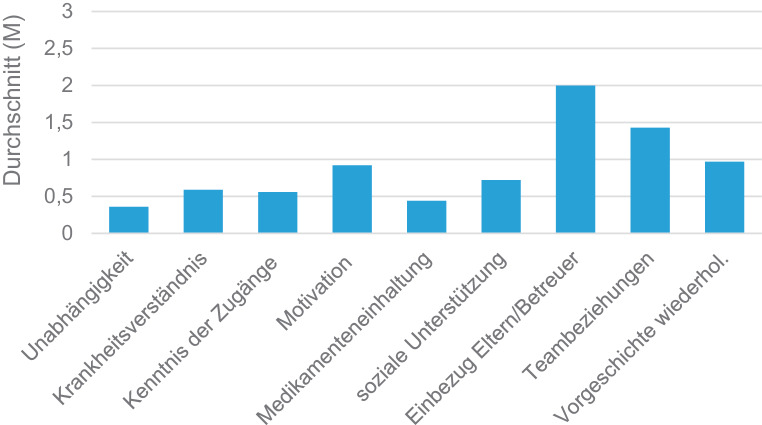
Transitionsbarrieren

Die Anzahl moderat bis schwerer Barrieren belief sich durchschnittlich auf 2,3 (M = 2,26; SD = 1,55). Die drei Transitionsbarrieren, welche am stärksten beeinträchtigt waren, aufgezählt in absteigender Reihenfolge waren: keine Beteiligung von Eltern/Betreuer:innen in der Behandlung (M = 2,00; SD = 0,90) zu wünschen, Schwierigkeiten beim Aufbau der Beziehungen zum Betreuungsteam (M = 1,43; SD = 0,86) und Schwierigkeiten beim Wiedergeben ihrer Krankengeschichte (M = 0,97; SD = 0,93) zu haben. Der von den Teilnehmenden durchschnittlich erreichte Wert in den einzelnen Bereichen wird in der Abb. 4 veranschaulicht.

#### Allgemeiner Krankheitsstatus

Anhand der Sub-Skala B schätzten die Adoleszenten ihren allgemeinen Krankheitsstatus ein. So gab die Mehrheit (N = 14; 35,9 %) an, leicht krank zu sein. 9 Personen (23,1 %) sahen sich als mäßig krank und 8 (20,5 %) als genesen an. Demgegenüber waren 7 Personen darunter, die ihren Krankheitsstatus als ernsthaft krank einstuften. Während eine Person (N = 1; 2,6 %) unter laufender Behandlung genesen sei. Keiner der Befragten beurteilte den eigenen Krankheitsstatus als sehr schwer krank.

#### Ausmaß und Qualität der Versorgung

##### A10: Qualität der Behandlung

Der überwiegende Großteil fand die erhaltene Versorgung entweder einigermaßen hilfreich (N = 17; 43,6 %) oder sehr hilfreich (N = 14; 35,9 %). Die aktuelle Versorgung war für ca. 10 % (N = 4; 10,3 %) eingeschränkt hilfreich und für weitere 10 % (N = 4; 10,3 %) war die Versorgung überhaupt nicht hilfreich, resultierend in einer Verschlechterung der Erkrankung.

##### A11: Ausmaß der Behandlung

Viele (N = 26; 66,7 %) betrachteten das Ausmaß ihrer aktuellen Versorgung als angemessen. Fast ein Viertel (N = 9; 23,1 %) sahen ihr Behandlungsteam nicht oft genug. Während 8 % (N = 3; 7,7 %) ihr Behandlungsteam zu oft treffen würden. Eine Person (2,6 %) gab an, sie traf ihr Behandlungsteam nicht oft genug, wodurch dies Auswirkungen auf ihren Krankheitszustand haben würde. Keiner der Teilnehmenden erklärte, das Behandlungsteam zu oft zu sehen mit Auswirkungen auf deren Arbeit, Ausbildung oder soziales Leben.

#### Symptomatik und die Symptomatik beeinflussenden Faktoren

**Abb. 5:**
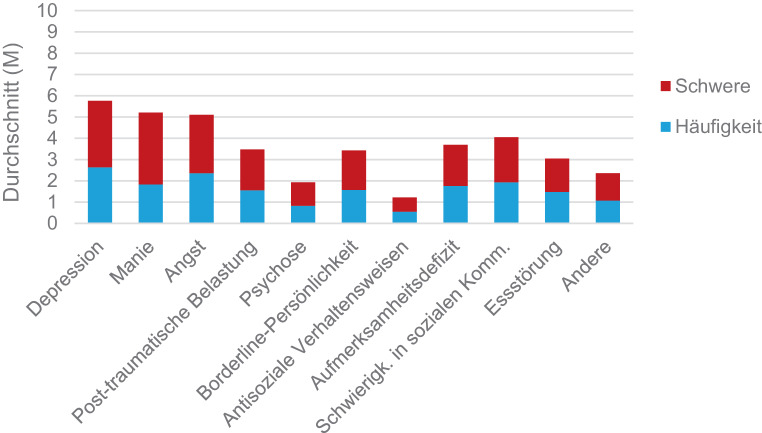
Häufigkeit und Schwere der Symptomatik

Mit Hilfe der Sub-Skala A wurde die Symptomatik mittels Selbsteinschätzungen der Adoleszenten erfragt. In der Abb. 5 wird die Häufigkeit und Schwere der Symptomausprägungen im Durchschnitt illustriert.

Basierend auf den Häufigkeitsangaben der Sub-Skala A, beschrieben 22 Teilnehmende (56,4 %), depressive Symptome „oft“ bis „die ganze Zeit“ (Skalenbereich 3–5) zu erleben. Wird der gleiche Skalenbereich herangezogen, präsentierten sich 20 Personen (51,3 %) mit ängstlichen Symptomen und 16 Personen (41,0 %) gaben Schwierigkeiten in der sozialen Kommunikation an. Weiters berichteten jeweils 14 Personen (35,9 %) von Aufmerksamkeitsdefiziten bzw. manischen Symptomen. Insgesamt haben 13 der befragten Adoleszenten (33,3 %) bereits einmal Erfahrungen mit Psychosen gemacht.

#### Risikofaktoren

**Abb. 6:**
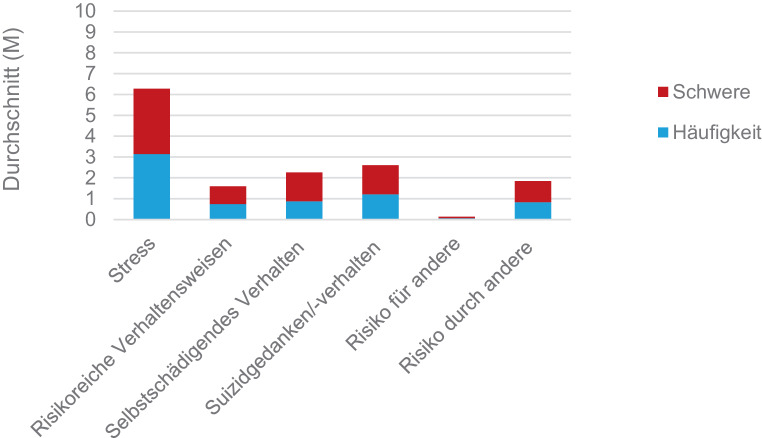
Häufigkeit und Schwere der Risikofaktoren

Die Abb. 6 zeigt die durchschnittlichen Werte zu den Risikofaktoren in ihrer Häufigkeit und Schwere. In diesem Zusammenhang wurde Stress (M = 6,26; SD 3,02) am häufigsten und schwersten erlebt.

In Bezug auf die Risikofaktoren war zudem erkennbar, dass 46,2 % der jungen Menschen (N = 18) Suizidgedanken bzw. -verhalten „selten“ bis „die ganze Zeit“ (Skalenbereich 1–5) gehabt haben. Selbstschädigende Verhaltensweisen haben 43,6 % (N = 17) entweder „selten“, „manchmal“ oder „oft“ erlebt. Während keiner der Teilnehmenden diese „fast die ganze Zeit“ oder „die ganze Zeit“ erfahren hat.

#### Funktionsniveau

**Abb. 7:**
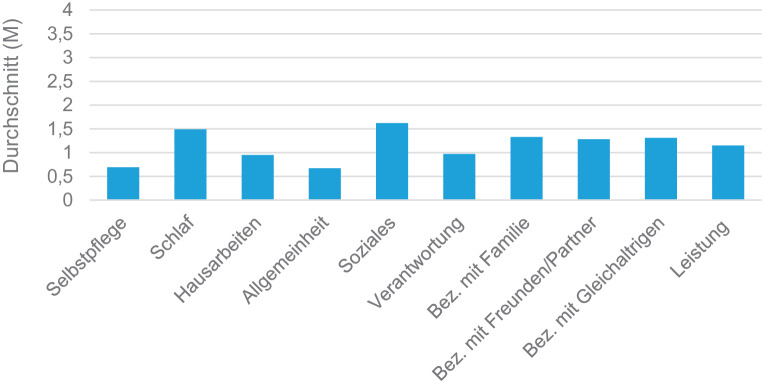
Funktionsniveau

Zur Darstellung des Funktionsniveaus der Teilnehmenden wurde die Sub-Skala C verwendet und in Abb. 7 als Balkendiagramm abgebildet. Die Bereiche Soziales (M = 1,62; SD = 1,29), Schlaf (M = 1,49; SD = 1,12) und Beziehungen mit Familienmitgliedern (M = 1,33; SD = 1,24) waren mit einer teilweisen Beeinträchtigung am häufigsten betroffen.

#### Zusammenhang zwischen der/dem Transitionsbereitschaft/-bedarf und der Identitätsentwicklung

**Abb. 8:**
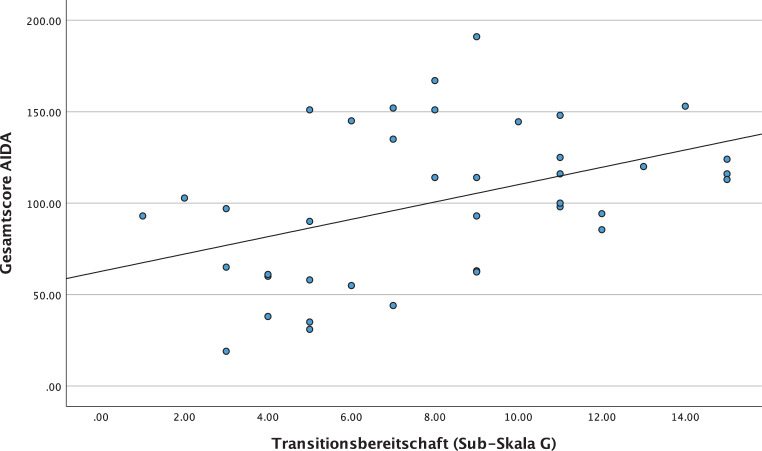
Korrelation zwischen der Transitionsbereitschaft und Identitätsentwicklung

Um Aussagen über den Zusammenhang zwischen der Transitionsbereitschaft und der Identitätsentwicklung treffen zu können, wurde der Gesamtscore des AIDA mit der Sub-Skala G aus dem TRAM in Korrelation nach Pearson gesetzt. Die grafische Darstellung der Korrelation erfolgt als Streudiagramm in Abb. 8. Die Berechnung ergab einen signifikanten Zusammenhang (*p* = 0,003) mit einer Effektstärke von r = 0,431.

**Abb. 9:**
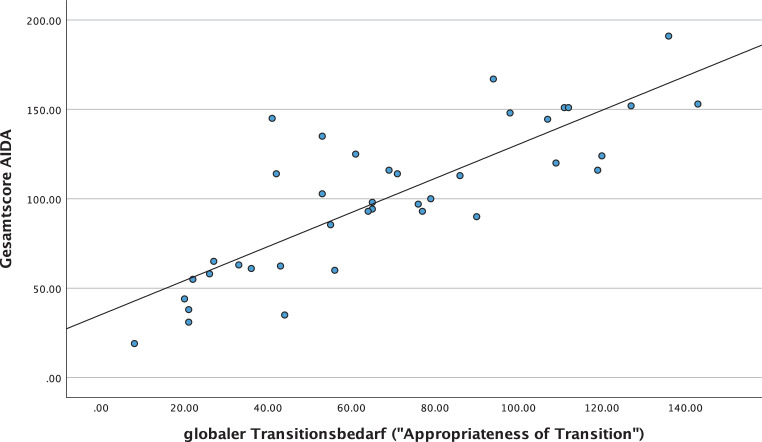
Korrelation zwischen dem Transitionsbedarf und der Identitätsentwicklung

In der Korrelationsanalyse nach Pearson wurde der Gesamtscore des AIDA mit dem globalen Transitionsbedarf („Appropriateness of Transition“) gleichgesetzt und berechnet. Dadurch konnte gezeigt werden, dass die Identitätsentwicklung signifikant mit dem Transitionsbedarf zusammenhängt (r = 0,821; *p* = < 0,001). Das bedeutet, eine diffuse Identitätsentwicklung wird mit einem erhöhten Transitionsbedarf assoziiert. Das Streudiagramm in der Abb. 9 verdeutlicht den linear positiven Zusammenhang zwischen den berechneten Variablen.

## Diskussion

Die vorliegende Studie gewährt einen ersten Überblick über die transitionsspezifischen Merkmale und Bedürfnisse von Patient:innen im Transitionsalter in Österreich. Außerdem wurden die Zusammenhänge zwischen der Identitätsentwicklung und der Transitionsbereitschaft bzw. des -bedarfs erfasst. Dadurch können Implikationen einer zukünftigen Transitionspsychiatrie abgeleitet werden.

An der vorliegenden Untersuchung nahmen adoleszente Patient:innen im Transitionsalter zwischen 17 bis 23 Jahren teil, die vorwiegend Symptome wie Depressionen, Ängste, Manien, Aufmerksamkeitsprobleme und Schwierigkeiten in der sozialen Kommunikation aufwiesen.

Dabei wurden internalisierende Symptome häufiger von weiblichen Personen erlebt, während männliche Teilnehmer öfter von externalisierenden Symptomen betroffen waren. Diese Verteilung lässt sich gleichermaßen in anderen Studien finden [22–25], bei denen Angsterkrankungen, Störungen des Sozialverhaltens, neurologische Entwicklungsstörungen und Depressionen ebenfalls als häufigste Störungen bei Kindern und Jugendlichen verzeichnet wurden.

Darüber hinaus gaben 33 % der Befragten der hier dargestellten Erhebung an, schon einmal psychotische Erfahrungen gemacht zu haben, ca. 44 % schilderten Selbstverletzungen und 46 % berichteten über Suizidgedanken bzw. -verhalten. Diese Ergebnisse stimmen ebenfalls mit denen anderer Erhebungen überein. Demnach wurden bei einer klinischen Population an Adoleszenten ähnlich hohe Werte von 30 bis 54 % für suizidale bzw. selbstverletzende Handlungen und 21 bis 33 % für psychotische Erfahrungen festgestellt [23, 26, 27]. Auch die beschriebenen Einschränkungen des Funktionsniveaus (vor allem Soziales, Schlaf und Beziehungen mit Familienmitgliedern) lassen sich in vergleichbare Untersuchungen einordnen [16, 23], welche die Förderung von sozialen Kompetenzen und Selbstmanagementfähigkeiten als eine der Kernaufgaben der transitionsspezifischen Versorgung definieren [28–30].

Hinsichtlich der Transitionsbereitschaft und -barrieren ging aus der umfassenden EU-weiten Milestone-Studie [12] hervor, dass insbesondere ein unzureichendes Wissen über geeignete Anlaufstellen, ein fehlendes Bewusstsein für die eigene Erkrankung als auch eine mangelnde Motivation, Hilfe zu suchen für einen vorzeitigen Therapieabbruch oder eine Ablehnung ursächlich sind [31, 32]. Gleichzeitig wurden bei einem erforderlichen Behandlungswechsel vertraute therapeutische Beziehungen aufgegeben. Durch eine unzureichende Zusammenarbeit zwischen KJP und EP mussten Adoleszente ihre Krankengeschichte teilweise an verschiedene Spezialisten weitergeben [15, 31]. Gleiches spiegeln die Ergebnisse der vorliegenden Untersuchung wider: Die hinderlichen Faktoren einer erfolgreichen Transition äußerten sich bei den befragten Patient:innen vorrangig darin, keine Einbeziehung der Eltern bzw. Betreuer:innen zu wünschen, Schwierigkeiten beim Aufbau von Beziehungen mit dem Behandlungsteam zu haben und die Krankengeschichte wiederholt wiedergeben zu müssen. Schließlich empfanden fast 23 % das Ausmaß ihrer Behandlung als zu gering und ca. 21 % waren mit der Hilfe, die sie in der KJP erhalten hatten, unzufrieden. Viele Jugendliche fühlen sich jedoch bereit, Entscheidungen eigenständig zu treffen, meist ohne Mithilfe der Eltern [23]. Dahingehend sehen Transitionsprogramme vor, gerade junge Menschen im Transitionsalter hinsichtlich ihrer Autonomie- und Selbstwirksamkeitsfähigkeiten gezielt zu fördern. Um sie in diesen Fähigkeiten zu unterstützen, kann eine Einführung in die Kompetenzen eines Erwachsenen (z. B. Steuererklärungen, Bewerbungsgespräche, Job- und Wohnmöglichkeiten, Sozialleistungen) ein erster Schritt sein [29].

Da der Aufbau einer stabilen Identität zu den zentralen Entwicklungsaufgaben von Adoleszenten zählt [1–3, 6, 15], wurde in der vorliegenden Studie auch der Zusammenhang zwischen der Identitätsentwicklung und der Transitionsbereitschaft bzw. des -bedarfs untersucht. Es konnte deutlich gemacht werden, dass eine beeinträchtigte Identitätsentwicklung mit einer geringeren Bereitschaft für eine erwachsenenpsychiatrische Behandlung assoziiert ist. Darüber hinaus stand eine nicht ausgereifte Identitätsentwicklung in Verbindung, einen erhöhten Bedarf einer Weiterversorgung zu zeigen. Da eine beeinträchtigte Identitätsentwicklung häufig Grundlage für die Entwicklung schwerwiegender Persönlichkeitspathologien darstellt, scheint es gerade bei Jugendlichen mit einer beeinträchtigten Identitätsentwicklung von Bedeutung, im Rahmen der Transition therapeutische Prozesse mit standardisierten Übergaben an die Weiterbehandler:innen aufrechtzuerhalten. Dadurch könnte sich das erhöhte Risiko von Behandlungsabbrüchen und Chronifizierungen deutlich reduzieren. Darüber hinaus sollten transitionspsychiatrische Behandlungskonzepte identitätsrelevante Themen und damit zusammenhängende Faktoren (wie z. B. Emotionsregulation, Beziehungsfähigkeit) mit beinhalten. Bereits etablierte Therapiemodelle wie die Dialektisch-Behaviorale-Therapie [33] oder das Adolescence-Identity-Treatment [34] könnten in die transitionsspezifische Behandlung integriert werden.

Um Adoleszenten im Transitionsalter in Österreich eine adäquate Versorgung zu ermöglichen, sollten also neben den von Singh et al. [10] beschriebenen strukturellen Möglichkeiten (Einbezug der Jugendlichen, Informationsaustausch, parallele Arbeit von aktuellen und neuen Behandlern, Planung vor und nach dem Übergang, Behandlungskontinuität), zugleich Strategien zur Verbesserung von patientenbezogenen Faktoren (z. B. Krankheitsbewusstsein, Selbstwirksamkeit, soziale Fähigkeiten, Identitätsentwicklung und Emotionsregulation) berücksichtigt werden. Der Einsatz standardisierter Messinstrumente wie der TRAM-Fragebogen kann dazu beitragen, die individuellen Transitionsbedürfnisse zu erfassen und adäquate (Weiter‑) Behandlungen zu planen. Dementsprechend bietet das in den USA entwickelte *Steps to care Model* (STCM) [35] einen Übergangsplan für den Eintritt in die erwachsenenpsychiatrische Behandlung unter Berücksichtigung dieser Faktoren. Die in Australien begründeten sogenannten „Headspace-Zentren“ gewähren Zugang zu diagnose-, alters- und fächerübergreifenden Behandlungsmodellen und wurden mittlerweile ebenfalls in anderen Ländern wie dem Soulspace-Berlin oder dem RECOVER-Hamburg implementiert [36, 37]. Zusätzlich kann der verstärkte Einsatz von digitalen Methoden (u. a. digitale Diagnostik, digitale Selbsthilfeprogramme, geführte online Therapien, Video-Gruppentherapien) zur Schließung von Versorgungslücken beitragen [29, 37]. So wurde auch die in Deutschland im Rahmen des Projekts „ProTransition“ entstandene App für junge Menschen in der Transitionsphase zur Förderung der Transitionsbereitschaft, des Selbstmanagements und der Selbstwirksamkeit entwickelt und in einer randomisiert-kontrollierten Studie überprüft [29]. Die bisher existierenden transitionspsychiatrischen Stationen in Österreich wie beispielsweise die transitionspsychiatrische Station der Klinik Hietzing in Wien oder die Adoleszenz-Tagesklinik in Innsbruck sollten weiter ausgebaut und auf andere Standorte übertragen werden [38]. Eine wichtige Schlüsselrolle könnte auch die Forschungsgruppe DOT („Die offene Tür“) der Ludwig Boltzmann Gesellschaft (LBG) und der Karl Landsteiner Privatuniversität (KL), als Österreichs derzeit einzigartiges Forschungszentrum für Transitionspsychiatrie, einnehmen [39]. Auch Weiterbildungsangebote sollten Adoleszenten spezifische Module beinhalten.

Als Fazit ergibt sich daraus, dass eine Integration von standardisierten Messinstrumenten in institutionelle Versorgungssysteme, die die transitionsbezogenen Bedürfnisse, die Transitionsbereitschaft und den -bedarf von jungen Menschen im Transitionsalter individuell erfassen, eine zielgerichtete und bedürfnisgerechte Behandlung bzw. Transition erleichtern kann. Auch eine interdisziplinäre Zusammenarbeit der Kinder- und Jugendpsychiatrie und der Erwachsenenpsychiatrie sowie eine kontinuierliche Überführung der therapeutischen Beziehungen im Transitionsprozess sollten standardisiert gewährleistet werden.

## Limitationen

Die Limitationen der vorliegenden Erhebung bestehen vor allem in der geringen Stichprobengröße, welche sich von 300 auf 39 Patien:innen reduzierte. Dadurch wurde die Repräsentativität der Studie stark eingeschränkt. Des Weiteren wurde die Selektion der Patient:innen nicht zufällig getätigt, sondern anhand einer Telefonliste durchgeführt, wobei es zu selektiven Drop-Outs aufgrund fehlender Werte in den Fragebögen und fehlenden Einverständniserklärungen kam. Eine zusätzliche Limitation dieser Untersuchung war die schwierige Rekrutierung der Adoleszenten, die zum Teil nicht (mehr) erreichbar waren oder eine Teilnahme aufgrund von fehlendem Interesse, zeitlichen Gründen bzw. aufgrund des Fragenformats (telefonisch gestützte Online-Befragung) ablehnten. Jugendliche mit einem hohen Schweregrad ihrer Erkrankung nahmen nicht teil (sehr schwer Kranke: N = 0; 0,0 %), wodurch es möglicherweise zu einem höheren Anteil an Patienten kam, die eine aktuelle Behandlungszufriedenheit aufweisen und keine Behandlung mehr benötigen (72 %). Zukünftige Studien sollten daher auch Akutpatienten mit stationären Aufenthalten und chronischen psychischen Erkrankungen einschließen. Außerdem gaben vermehrt weibliche Teilnehmer:innen (N = 27; 69,2 %) ihr Einverständnis zur Teilnahme an der Studie, wodurch internalisierende Störungen vermutlich häufiger auftraten. Auch Aspekte der sozialen Erwünschtheit während der Beantwortung der Fragebögen müssen bei den Limitationen berücksichtigt werden.

## Interessenkonflikt

K. Kunczicky, A.-C. Jahnke-Majorkovits und K. Sevecke geben an, dass kein Interessenkonflikt besteht. Die Autoren versichern, dass diese Arbeit ihr geistiges Eigentum ist und dass sie das Copyright besitzen. Des Weiteren erklären die Autoren, dass bisher weder das ganze Manuskript noch Teile davon in deutscher oder englischer Sprache publiziert bzw. in anderen deutschsprachigen Zeitschriften eingereicht worden sind.

## References

[1] Fegert JM, Freyberger HJ. Adoleszenz eine Lebensphase weitet sich aus. Herausforderungen an eine Psychologie und Psychopathologie des Transitionsalters. PiD. 2017;18(02):16–22. 10.1055/s-0043-103880. 10.1055/s-0043-103880

[2] Arnett JJ, Žukauskienė R, Sugimura K. The new life stage of emerging adulthood at ages 18–29 years: implications for mental health. Lancet Psychiatry. 2014;1(7):569–76. 10.1016/S2215-0366(14)00080-7. 26361316 10.1016/S2215-0366(14)00080-7

[3] Seiffge-Krenke I. „Emerging Adulthood“: Forschungsbefunde zu objektiven Markern, Entwicklungsaufgaben und Entwicklungsrisiken. ZPPP. 2015;63(3):165–74. 10.1024/1661-4747/a000236. 10.1024/1661-4747/a000236

[4] Lambert M, Bock T, Naber D, Löwe B, Schulte-Markwort M, Schäfer I, et al. Die psychische Gesundheit von Kindern, Jugendlichen und jungen Erwachsenen – Teil 1: Häufigkeit, Störungspersistenz, Belastungsfaktoren, Service-Inanspruchnahme und Behandlungsverzögerung mit Konsequenzen. Fortschr Neurol Psychiatr. 2013;81(11):614–27. 10.1055/s-0033-1355843. 24194055 10.1055/s-0033-1355843

[5] Wagner G, Zeiler M, Waldherr K, Philipp J, Truttmann S, Dür W, et al. Mental health problems in Austrian adolescents: a nationwide, two-stage epidemiological study applying DSM‑5 criteria. Eur Child Adolesc Psychiatry. 2017;26(12):1483–99. 28540609 10.1007/s00787-017-0999-6 PMC5701961

[6] Erikson EH. Identity: Youth and crisis. New York: Norton; 1968.

[7] Kernberg OF. The diagnosis of borderline conditions in adolescence. Adolesc Psychiatry. 1978;6:298–319. 742674

[8] Reneses B, Escudero A, Tur N, Agüera-Ortiz L, Moreno DM, Saiz-Ruiz J, et al. The black hole of the transition process: dropout of care before transition age in adolescents. Eur Child Adolesc Psychiatry. 2022; 10.1007/s00787-021-01939-8. 35048161 10.1007/s00787-021-01939-8 PMC10276128

[9] Signorini G, Singh SP, Marsanic VB, Dieleman G, Dodig-Ćurković K, Franic T, et al. The interface between child/adolescent and adult mental health services: results from a European 28-country survey. Eur Child Adolesc Psychiatry. 2018;27(4):501–11. 10.1007/s00787-018-1112-5. 29368253 10.1007/s00787-018-1112-5

[10] Singh SP, Paul M, Ford T, Kramer T, Weaver T, McLaren S, et al. Process,outcomeand experience of transition from child to adult mental healthcare: multiperspective study. Br J Psychiatry. 2010;197(4):305–12. 10.1192/bjp.bp.109.075135. 20884954 10.1192/bjp.bp.109.075135

[11] Loos S, Walia N, Becker T, Puschner B. Lost in transition? Perceptions of health care among young people with mental health problems in Germany: a qualitative study. Child Adolesc Psychiatry Ment Health. 2018;12:41. 10.1186/s13034-018-0249-9. 30093915 10.1186/s13034-018-0249-9 PMC6080358

[12] Tuomainen H, Schulze U, Warwick J, Paul M, Dieleman GC, Franić T, et al. Managing the link and strengthening transition from child to adult mental health Care in Europe (MILESTONE): background, rationale and methodology. BMC Psychiatry. 2018;18(1):167. 10.1186/s12888-018-1758-z. 29866202 10.1186/s12888-018-1758-z PMC5987458

[13] Singh SP, Tuomainen H, Bouliotis G, Canaway A, De Girolamo G, Dieleman G, et al. Effect of managed transition on mental health outcomes for young people at the child-adult mental health service boundary: a randomised clinical trial. Psychol Med. 2021. 10.1017/S0033291721003901. 37310306 10.1017/S0033291721003901 PMC10123823

[14] Pollak E, Kapusta ND, Diehm R, Plener PL, Skala K. Transitions- und Adoleszenzpsychiatrie in Österreich: Eine Pilotuntersuchung zur Sicht von Expert(innen). Z Kinder Jugendpsychiatr Psychother. 2018;46(4):325–35. 10.1024/1422-4917/a000559. 29183258 10.1024/1422-4917/a000559

[15] Kunczicky K. Transitionspsychiatrische Bedürfnisse von Adoleszenten und der Zusammenhang mit der Identitätsentwicklung. 2023. Medizinische Universität Innsbruck: Diplomarbeit.

[16] Santosh P, Singh J, Adams L, Mastroianni M, Heaney N, Lievesley K, et al. Validation of the transition readiness and appropriateness measure(TRAM) for the managing the link and strengthening transition from child to adult mental Healthcare ineurope (MILESTONE) study. BMJ Open. 2020;10(6):e33324. 10.1136/bmjopen-2019-033324. 32580979 10.1136/bmjopen-2019-033324 PMC7312331

[17] Goth K, Schmeck K. Das Inventar AIDA (Assessment of Identity Development in Adolescence) Deutschsprachige Version: Ein Fragebogen zur Selbstbeantwortung für die Erfassung der Identitätsentwicklung im Jugendalter – Kurzmanual. Offenbach: academic-tests. 2018. https://academic-tests.com/aida/. Zugegriffen: 20. Apr. 2023.

[18] Goth K, Foelsch P, Schlüter-Müller S, Birkhölzer M, Jung E, Pick O, et al. Assessment of identity development and identity diffusion in adolescence—Theoretical basis and psychometric properties of the self-report questionnaire AIDA. Psychiatry Ment Health. 2012;6(1):27. 10.1186/1753-2000-6-27. 10.1186/1753-2000-6-27 PMC3485126 22812911

[19] González Flores S, Goth K, Díaz-Hernandez RA. Psychometric properties of a cultural adapted version of the assessment of identity development in adolescence in Panama. Front Psychiatry. 2022;13:806033. 10.3389/fpsyt.2022.806033. 35432021 10.3389/fpsyt.2022.806033 PMC9009042

[20] Sharp C, McLaren V, Musetti A, Vanwoerden S, Hernandez Ortiz J, Schmeck K, et al. The assessment of identity development in adolescence (AIDA) questionnaire: first psychometric evaluation in two North American samples of young people. J Pers Assess. 2022; 10.1080/00223891.2022.2119860. 36121311 10.1080/00223891.2022.2119860

[21] Bortz J, Schuster C. Statistik für Human- und Sozialwissenschaftler. Berlin, Heidelberg: Springer; 2011.

[22] Fuchs M, Karwautz A. Epidemiologie psychischer Störungen bei Kindern und Jugendlichen: Eine narrative Übersichtsarbeit unter Berücksichtigung österreichischer Daten. neuropsychiatrie. 2017;31(3):96–102. 10.1007/s40211-017-0238-x. 28853032 10.1007/s40211-017-0238-x

[23] Gerritsen SE, Maras A, van Bodegom LS, Overbeek MM, Verhulst FC, Wolke D, et al. Cohort profile: demographic and clinical characteristics of the MILESTONE longitudinal cohort of young people approaching the upper age limit of their child mental health care service in Europe. BMJ Open. 2021;11(12):e53373. 10.1136/bmjopen-2021-053373. 34916319 10.1136/bmjopen-2021-053373 PMC8679118

[24] Polanczyk GV, Salum GA, Sugaya LS, Caye A, Rohde LA. Annual research review: a meta-analysis of the worldwide prevalence of mental disorders in children and adolescents. J Child Psychol Psychiatry. 2015;56(3):345–65. 10.1111/jcpp.12381. 25649325 10.1111/jcpp.12381

[25] Ravens-Sieberer U, Wille N, Erhart M, Bettge S, Wittchen HU, Rothenberger A. Prevalence of mental health problems among children and adolescents in Germany: results of the BELLA study within the National Health Interview and Examination Survey. Eur Child Adolesc Psychiatry. 2008;17(1):22–33. 10.1007/s00787-008-1003-2. 19132301 10.1007/s00787-008-1003-2

[26] Kaess M, Parzer P, Mattern M, Plener PL, Bifulco A, Resch F, et al. Adverse childhood experiences and their impact on frequency, severity, and the individual function of nonsuicidal self-injury in youth. Psychiatry Res. 2013;206(2–3):265–72. 10.1016/j.psychres.2012.10.012. 23159195 10.1016/j.psychres.2012.10.012

[27] Karow A, Lipp M, Schweigert E, Sengutta M, Wiltfang G, Wittmann L, et al. Alters-, diagnose- und fachübergreifende stationäre Behandlung für Jugendliche und junge Erwachsene (16–25 Jahre) in der Adoleszenzpsychiatrie. Psychiatr Prax. 2018;45(5):248–55. 10.1055/s-0043-120249. 29237195 10.1055/s-0043-120249

[28] Abzieher P, Lipp M, Staats JH, Banaschewski T, Driessen M, Karow A. Behandlungsangebote der Adoleszenzpsychiatrie – Ergebnisse einer deutschlandweiten Erhebung. Fortschr Neurol Psychiatr. 2019;87(11):645–52. 10.1055/a-1011-4198. 31756743 10.1055/a-1011-4198

[29] Ilgaz A, Fegert JM, Schulze UME, Baumeister H. Unterstützungsmöglichkeiten von jungen Erwachsenen mit einer psychischen Erkrankung und Herausforderungen während der Transition. Nervenheilkunde. 2022;41(09):560–8. 10.1055/a-1824-7839. 10.1055/a-1824-7839

[30] Mehler-Wex C. Syndromspezifische und ganzheitliche Adoleszenzpsychiatrie. Reifungskrisen – Teil 2: Folgen und Lösungen. NeuroTransmitter. 2013;24(7–8):36–42. 10.1007/s15016-013-0267-z

[31] Broad KL, Sandhu VK, Sunderji N, Charach A. Youth experiences of transition from child mental health services to adult mental health services: a qualitative thematic synthesis. BMC Psychiatry. 2017;17(1):380. 10.1186/s12888-017-1538-1. 29183289 10.1186/s12888-017-1538-1 PMC5706294

[32] Radez J, Reardon T, Creswell C, Lawrence PJ, Evdoka-Burton G, Waite P. Why do children and adolescents (not) seek and access professional help for their mental health problems? A systematic review of quantitative and qualitative studies. Eur Child Adolesc Psychiatry. 2021;30(2):183–211. 10.1007/s00787-019-01469-4. 31965309 10.1007/s00787-019-01469-4 PMC7932953

[33] Miller AL. Dialectical behavior therapy: a new treatment approach for suicidal adolescents. Am J Psychother. 1999;53(3):413–7. 10.1176/appi.psychotherapy.1999.53.3.413. 10586303 10.1176/appi.psychotherapy.1999.53.3.413

[34] Clarkin JF, Cain NM, Lenzenweger MF. Advances in transference-focused psychotherapy derived from the study of borderline personality disorder: clinical insights with a focus on mechanism. Curr Opin Psychol. 2018;21:80–5. 10.1016/j.copsyc.2017.09.008. 29065381 10.1016/j.copsyc.2017.09.008

[35] Farrell ML. Transitioning adolescent mental health care services: The steps to care model. J Child Adolesc Psychiatr Nurs. 2022;35(4):301–6. 10.1111/jcap.12377. 35383413 10.1111/jcap.12377

[36] McGorry P, Trethowan J, Rickwood D. Creating headspace for integrated youth mental health care. World Psychiatry. 2019;18(2):140–1. 10.1002/wps.20619. 31059618 10.1002/wps.20619 PMC6502425

[37] Lambert M, Karow A, Gallinat J, Lüdecke D, Kraft V, Rohenkohl A, et al. Study protocol for a randomised controlled trial evaluating an evidence-based, stepped and coordinated care service model for mental disorders (RECOVER). BMJ Open. 2020;10(5):e36021. 10.1136/bmjopen-2019-036021. 32371520 10.1136/bmjopen-2019-036021 PMC7223141

[38] Fliedl R, Ecker B, Karwautz A. Kinder- und jugendpsychiatrische Versorgung 2019 in Österreich – Stufen der Versorgung, Ist-Stand und Ausblick. Neuropsychiatr. 2020;34(4):179–88. 10.1007/s40211-020-00374-6. 33258039 10.1007/s40211-020-00374-6 PMC7732792

[39] Höflich A, Schrank B, Aigner M. Herausforderung Transitionspsychiatrie im Rahmen der Erwachsenenpsychiatrie. Psychoprax Neuroprax. 2022;25:284–8. 10.1007/s00739-022-00825-5. 10.1007/s00739-022-00825-5

